# Supplementary material for: Iterative improvement in the automatic modular design of robot swarms
Source: PeerJ Comput Sci. 2020 Dec 7;6:e322. doi: 10.7717/peerj-cs.322 (PMC7924708; doi:10.7717/peerj-cs.322)
Supplement: Supplemental Information 3 [file peerj-cs-06-322-s003.zip › argos3/doc/api/standalone/a00642_source.html]

ARGoS: plugins/simulator/visualizations/qt-opengl/qtopengl\_lua\_editor.cpp Source File


- Main Page
- Related Pages
- Namespaces
- Classes
- Files

- File List
- File Members

# plugins/simulator/visualizations/qt-opengl/qtopengl\_lua\_editor.cpp

Go to the documentation of this file.

```
00001 
00007 #include "qtopengl_lua_editor.h"
00008 #include "qtopengl_lua_syntax_highlighter.h"
00009 
00010 #include <QPainter>
00011 #include <QTextBlock>
00012 
00013 namespace argos {
00014 
00015    /****************************************/
00016    /****************************************/
00017 
00018    CQTOpenGLLuaEditor::CQTOpenGLLuaEditor(QWidget* pc_parent) :
00019       QPlainTextEdit(pc_parent) {
00020       /* Set font */
00021       QFont cFont;
00022       cFont.setFamily("Monospace");
00023       cFont.setStyleHint(QFont::Monospace);
00024       cFont.setFixedPitch(true);
00025       setFont(cFont);
00026       /* Set tab width to 3 */
00027       QFontMetrics cFontMetrics(cFont);
00028       setTabStopWidth(3 * cFontMetrics.width(' '));
00029       /* Set syntax highlighting */
00030       new CQTOpenGLLuaSyntaxHighlighter(document());
00031       /* Set line numbering */
00032       m_pcLineNumberArea = new CLineNumberArea(this);
00033       /* Connect signals */
00034       connect(this, SIGNAL(blockCountChanged(int)),
00035               this, SLOT(UpdateLineNumberAreaWidth(int)));
00036       connect(this, SIGNAL(updateRequest(const QRect&, int)),
00037               this, SLOT(UpdateLineNumberArea(const QRect&, int)));
00038       connect(this, SIGNAL(cursorPositionChanged()),
00039               this, SLOT(HighlightCurrentLine()));
00040       /* Final touches */
00041       UpdateLineNumberAreaWidth(0);
00042       HighlightCurrentLine();
00043    }
00044 
00045    /****************************************/
00046    /****************************************/
00047 
00048    void CQTOpenGLLuaEditor::LineNumberAreaPaintEvent(QPaintEvent* pc_event) {
00049       QPainter cPainter(m_pcLineNumberArea);
00050       cPainter.fillRect(pc_event->rect(), Qt::lightGray);
00051 
00052 
00053       QTextBlock cBlock = firstVisibleBlock();
00054       int nBlockNumber = cBlock.blockNumber();
00055       int nTop = (int) blockBoundingGeometry(cBlock).translated(contentOffset()).top();
00056       int nBottom = nTop + (int) blockBoundingRect(cBlock).height();
00057 
00058       while (cBlock.isValid() && nTop <= pc_event->rect().bottom()) {
00059          if (cBlock.isVisible() && nBottom >= pc_event->rect().top()) {
00060             QString strNumber = QString::number(nBlockNumber + 1);
00061             cPainter.setPen(Qt::black);
00062             cPainter.drawText(0, nTop,
00063                               m_pcLineNumberArea->width(), fontMetrics().height(),
00064                               Qt::AlignRight, strNumber);
00065          }
00066 
00067          cBlock = cBlock.next();
00068          nTop = nBottom;
00069          nBottom = nTop + (int) blockBoundingRect(cBlock).height();
00070          ++nBlockNumber;
00071       }
00072    }
00073 
00074    /****************************************/
00075    /****************************************/
00076 
00077    int CQTOpenGLLuaEditor::LineNumberAreaWidth() {
00078       int nDigits = 1;
00079       int nMax = qMax(1, blockCount());
00080       while (nMax >= 10) {
00081          nMax /= 10;
00082          ++nDigits;
00083       }
00084       int nSpace = 3 + fontMetrics().width(QLatin1Char('9')) * nDigits;
00085       return nSpace;
00086    }
00087 
00088    /****************************************/
00089    /****************************************/
00090 
00091    void CQTOpenGLLuaEditor::resizeEvent(QResizeEvent* pc_event) {
00092       QPlainTextEdit::resizeEvent(pc_event);
00093       QRect cRect = contentsRect();
00094       m_pcLineNumberArea->setGeometry(QRect(cRect.left(), cRect.top(),
00095                                             LineNumberAreaWidth(), cRect.height()));
00096    }
00097 
00098    /****************************************/
00099    /****************************************/
00100 
00101    void CQTOpenGLLuaEditor::UpdateLineNumberAreaWidth(int) {
00102       setViewportMargins(LineNumberAreaWidth(), 0, 0, 0);
00103    }
00104 
00105    /****************************************/
00106    /****************************************/
00107 
00108    void CQTOpenGLLuaEditor::HighlightCurrentLine() {
00109       QList<QTextEdit::ExtraSelection> cListExtraSel;
00110 
00111       if (!isReadOnly()) {
00112          QTextEdit::ExtraSelection cSel;
00113          QColor cLineColor = QColor(Qt::yellow).lighter(160);
00114          cSel.format.setBackground(cLineColor);
00115          cSel.format.setProperty(QTextFormat::FullWidthSelection, true);
00116          cSel.cursor = textCursor();
00117          cSel.cursor.clearSelection();
00118          cListExtraSel.append(cSel);
00119       }
00120       setExtraSelections(cListExtraSel);
00121    }
00122 
00123    /****************************************/
00124    /****************************************/
00125 
00126    void CQTOpenGLLuaEditor::UpdateLineNumberArea(const QRect& c_rect,
00127                                                  int n_dy) {
00128       if(n_dy) {
00129          m_pcLineNumberArea->scroll(0, n_dy);
00130       }
00131       else {
00132          m_pcLineNumberArea->update(0, c_rect.y(),
00133                                     m_pcLineNumberArea->width(), c_rect.height());
00134       }
00135       if(c_rect.contains(viewport()->rect())) {
00136          UpdateLineNumberAreaWidth(0);
00137       }
00138    }
00139    
00140    /****************************************/
00141    /****************************************/
00142    
00143 }
```

---

Generated on 10 Jul 2018 for ARGoS by 
 1.6.1
